# Is hospital discharge administrative data an appropriate source of information for cancer registries purposes? Some insights from four Spanish registries

**DOI:** 10.1186/1472-6963-10-9

**Published:** 2010-01-08

**Authors:** Enrique Bernal-Delgado E, Carmen Martos, Natalia Martínez, María Dolores Chirlaque, Mirari Márquez, Carmen Navarro, Lauro Hernando, Joaquín Palomar, Isabel Izarzugaza, Nerea Larrañaga, Olatz Mokoroa, M Cres Tobalina, Joseba Bidaurrazaga, María José Sánchez, Carmen Martínez, Miguel Rodríguez, Esther Pérez, Yoe Ling Chang

**Affiliations:** 1 Health Services Research Unit, Institute for Health Sciences in Aragon, Zaragoza, Spain; 2 Zaragoza Cancer Registry, Department of Health, Zaragoza, Spain; 3 Basque-Country Cancer Registry, Department of Health, Vitoria-Gasteiz, Spain; 4 Murcia Cancer Registry at Public Health Department, Regional Health Council, Murcia, Spain; 5 Planning and Health Financing Department, Regional Health Council, Murcia, Spain; 6 Granada Cancer Registry, Andalusian School of Public Health, Granada, Spain; 7 Network for Biomedical Research in Epidemiology and Public Health (CIBERESP), Madrid, Spain

## Abstract

**Background:**

The use of hospital discharge administrative data (HDAD) has been recommended for automating, improving, even substituting, population-based cancer registries. The frequency of false positive and false negative cases recommends local validation.

**Methods:**

The aim of this study was to detect newly diagnosed, false positive and false negative cases of cancer from hospital discharge claims, using four Spanish population-based cancer registries as the gold standard. Prostate cancer was used as a case study.

**Results:**

A total of 2286 incident cases of prostate cancer registered in 2000 were used for validation. In the most sensitive algorithm (that using five diagnostic codes), estimates for Sensitivity ranged from 14.5% (CI95% 10.3-19.6) to 45.7% (CI95% 41.4-50.1). In the most predictive algorithm (that using five diagnostic and five surgical codes) Positive Predictive Value estimates ranged from 55.9% (CI95% 42.4-68.8) to 74.3% (CI95% 67.0-80.6). The most frequent reason for false positive cases was the number of prevalent cases inadequately considered as newly diagnosed cancers, ranging from 61.1% to 82.3% of false positive cases. The most frequent reason for false negative cases was related to the number of cases not attended in hospital settings. In this case, figures ranged from 34.4% to 69.7% of false negative cases, in the most predictive algorithm.

**Conclusions:**

HDAD might be a helpful tool for cancer registries to reach their goals. The findings suggest that, for automating cancer registries, algorithms combining diagnoses and procedures are the best option. However, for cancer surveillance purposes, in those cancers like prostate cancer in which care is not only hospital-based, combining inpatient and outpatient information will be required.

## Background

Population-based cancer registries (hereinafter registries) are usually considered the main tool for cancer surveillance purposes. Unfortunately, there are some geographic areas which are not covered by a registry [1] and concerns about rates of underreporting remain current [2].

Although some improvements have been made in the way a registry deals with data, most of the activity is still managed manually. Additionally, registries gather information from different sources with a large variation in their reliability and validity. Finally, all these processes imply delays in the production of relevant information. Thus, automating all these processes has become a priority in order to improve cancer registries accuracy, efficiency and timeliness [3,4].

The use of secondary databases, particularly those which provide information on hospital discharges (i.e. hospital discharges administrative data [HDAD]), has been recommended for improving registries performance in terms of the number of incident cancer cases retrieved [2,5-7]; in some circumstances, they have even been used as a substitute for a registry [8,9]. However, the nature of the information (hospital-based data), the purposes for which it is recorded (not to act as a diseases registry) and the methodology (based on the information declared by the clinicians) require local validation.

In fact, some studies have shown variability in the capacity of HDAD to identify incident cancer cases. For example, depending on the definition used to name incident cases, the timeframe of retrieval, or whether the patient received surgical treatment, positive predictive values (PPV) ranged from 43% to 92% in breast cancer [10-12]. Other research, focusing on prostate, lung, colorectal, breast, pancreas and endometrial cancer in elderly Americans showed similar variation in estimates: sensitivity ranged from 46% in prostate cancer to 82% in colorectal cancer [13].

As a consequence of these results, positive and negative false causes have been described. So, prevalent cases, coding error misclassification (benign, in situ, etc.), multiple readmissions, coding cancer in a secondary diagnostic position or not having a concurrent surgical procedure in the same episode, have been suggested causes of false positive cases of incident cancer. Private hospitalizations, outpatient care, elderly people, patients with comorbidity or patients admitted for conservative procedures have been proposed -among others- as causes of negative false cases of incident cancer [14,15].

Unfortunately, few studies have focused on HDAD validity in the Spanish context. Recently, a study discussed this matter comparing colorectal cancer admissions with cases registered in a Spanish registry [16]. When only the first diagnostic code was considered, the sensitivity of the HDAD reached 80% with a positive predictive value of 75%. When all the coded diagnoses were considered, sensitivity rose to 85% reducing the PPV to 64%.

Finally, HDAD validity is expected to be different in those cases in which variability in medical practice is relevant. For example, colorectal cancer in which admission for surgery is the rule and prostate cancer, where options for treatment go from a watchful waiting strategy (ambulatory care) to a radical prostatectomy (hospital care) [17]. In fact, for prostatectomy on prostate cancer, a 7-fold variation was found between high and low intensity healthcare areas in Spain [18].

The aim of our study was to detect incident, false positive and false negative cases of cancer on hospital discharge claims, using four Spanish population-based cancer registries as the gold standard. Prostate cancer was used as a case study.

## Methods

### Design

We firstly carried out a linking-records-based validation study. Using a cross-section of 1998 to 2000 HDAD from 48 hospitals in 4 Spanish regions (Basque Country, Region of Murcia, and the provinces of Granada and Zaragoza), prostate cancer discharges in 2000 were studied. Public hospitals provided the information for the study, except in the case of Murcia where information from private hospitals was also included.

For validation purposes, the lack of an independent gold standard obliged us to use cancer registries, although some overlapping might be found because HDAD is usually a basic source of information for cancer registries. The four Spanish population-based registries, used as the gold standard in the study, cover a population of about 2.5 million inhabitants, and fulfil international standard procedures [19]. The Health Regional Authorities in Andalusia, Aragon, Basque Country and Region of Murcia, which are in charge of both the cancer registries and the HDAD, allowed its use for the purposes of this study.

### Linking-record process

**Figure 1:**
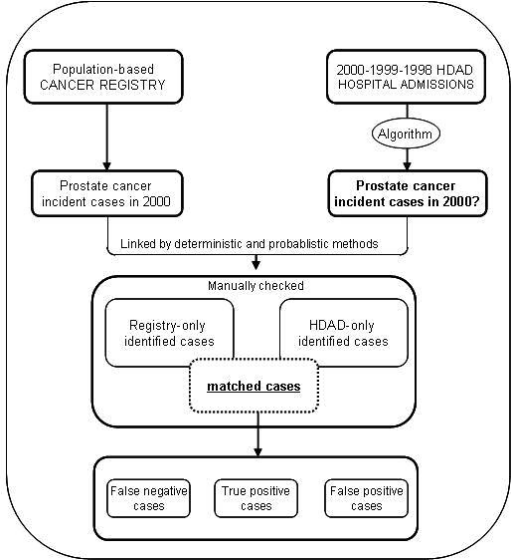
**Flowchart representing the linking record process**.

Once prostate cancer cases were retrieved from HDAD, a linking-record process was developed in order to match these cases with those identified as incident cases in 2000 by each local registry. The process basically consisted in matching -both in a deterministic or a probabilistic way- each prostate cancer case by hospital record ID, hospital of admission, date of birth, insurance identification number or name of patient [20]. All cases were checked after automatic linking process finished. (Figure 1)

As a consequence, four matched datasets were obtained from this process and used in the analyses: the Basque Country Dataset (BD), the Granada Dataset (GD), the Murcia Dataset (MD) and the Zaragoza Dataset (ZD).

### Main endpoints

HDAD validity for detecting incident cases of prostate cancer was assessed by estimating sensitivity, positive predictive value and analysing false negative and false positive underlying causes for the most sensitive (i.e., more helpful for cancer surveillance purposes) and the most predictive strategy of analysis (i.e., more useful for automating data).

### Definition of malignant prostate cancer incident case

The definition of a malignant prostate cancer was based on combinations of diagnostic and procedure codes according to the International Classification of Diseases, 9^th ^Revision, Clinical Modification ICD-9-CM (table 1)

**Table 1:** Codes defining malignant prostate neoplasm

	ICD 9th-CM codes
Inclusion Codes	***Diagnostic codes***
	185
	***Surgical codes***
	605 6069, 623 - 6242, 6029, 603, 604, 6061, 6062

Exclusion Codes	***Antecedent of cancer at the same site***
	V1046
	***Antecedent of episode of care for prostate cancer***
	V581, V580, V660, V661, V662, V670, V671, V672
	***Miscellaneous***
	V711

Among those selected "cases under study", both incident and prevalent cases were found. To reduce the possibility of retrieving prevalent cases (as opposed to incident ones), an active search was made in the two previous years (1998 and 1999). Those episodes with the same diagnostic code, metastases codes, and those with a code of cancer antecedent or episode of care for cancer were excluded.

As previous work suggests [11,13], in order to get a more specific definition, four different strategies (computing algorithms) combining both the number of codes and the information from diagnoses and procedures were checked. These four strategies were: 1) selecting episodes which had an eligible code in the main diagnostic position (Algorithm 1); 2) selecting episodes which had an eligible code in the first five diagnostic positions (Algorithm 2); 3) selecting episodes that had a code of cancer in the main diagnostic position and also a related surgical procedure (Algorithm 3); and 4) selecting episodes which had a code of cancer among the first five diagnostic positions and a related surgical procedure (Algorithm 4). When procedures were used (i.e. algorithm 3 and 4), five first positions were considered.

Therefore, once cancer codes were selected, possible prevalent cases were excluded by searching in the two previous years. Then, the four aforementioned strategies were checked to select the best in detecting "incident cancer cases".

### Analysis

Number and percentage of matched cases and causes of failure were analyzed. Among matched and unmatched cases, underlying causes of disagreement were studied; thus, false positive and false negative cases were recorded and discussed.

Finally, in order to determine the validity of the HDAD to detect incident cases of cancer, two indexes were estimated: 1) Sensitivity, as the percentage of cases considered incident cases in the HDAD within those true incident cases recorded in the registry; and, 2) the Positive Predictive Value as the percentage of true cases within those cases considered incident cases in the HDAD. Both indexes were calculated for each one of the four different strategies and 95% confidence intervals (CI95%) were estimated using an exact binomial test [21]. All analyses were conducted using STATA v9.

## Results

A total of 2286 cases of prostate cancer registered in 2000 were used. 231 cases were included from Granada cancer registry (crude rate of 57.8 per 10^5 ^inhabitants); 1150 cases from Basque Country cancer registry (crude rate of 113.4 per 10^5 ^inhabitants); 411 cases from Murcia cancer registry (crude rate of 71.4 per 10^5 ^inhabitants) and 494 cases from Zaragoza cancer registry (crude rate of 117.9 per 10^5 ^inhabitants). Table 2 compares these facts with the number of cases retrieved from the HDAD once algorithms were implemented.

**Table 2:** Sensitivity (S) and Positive Predictive Values (PPV). (by Algorithm and Region)

	Algorithm 1 Dx1	Algorithm 2 Dx1 to Dx5	Algorithm 3 Dx1&Pr1-Pr5	Algorithm 4 Dx1-Dx5&Pr1-Pr5
**Granada**				
Cases identified in Registry	231	231	231	231
Cases identified in HDAD	55	113	33	33
Sensitivity (CI95%)	12.9 (8.9-18.0)	14.5 (10.3-19.6)	8.7 (5.4-13.1)	8.7(5.4-13.1)
PPV (CI95%)	54.5 (40.6-68.0)	39.8 (30.7-49.5)	60.6 (42.1-77.1)	60.6 (42.1- 77.1)

**Basque Country**				
Cases identified in Registry	1150	1150	1150	1150
Cases identified in HDAD	372	632	245	250
Sensitivity (CI95%)	17.8 (15.6-20.1)	24.3 (21.8-26.8)	13.2 (11.3-15.3)	13.5 (11.5-15.6)
PPV (CI95%)	55.1 (49.9-60.2)	44.1 (40.2-48.1)	62.0 (55.6-68.1)	62.0 (55.6-68.0)

**Murcia**				
Cases identified in Registry	411	411	411	411
Cases identified in HDAD	118	218	56	59
Sensitivity (CI95%)	14.4(11.1-18.1)	23.6 (19.5-28.0)	7.3 (4.9-10.2)	8.0 (5.5-11.0)
PPV (CI95%)	50.0 (40.6-59.3)	44.5 (37.7-51.3)	53.6 (39.7-67.0)	55.9 (42.4-68.8)

**Zaragoza**				
Cases identified in Registry	494	494	494	494
Cases identified in HDAD	217	378	169	171
Sensitivity (CI95%)	30.4 (26.3-34.4)	45.7 (41.4-50.1)	25.3 (21.5-29.3)	25.7 (22.0-29.8)
PPV (CI95%)	69.1 (64.1-74.2)	59.8 (55.4-64.8)	74.0 (66.6-80.4)	74.3 (67.0-80.6)

S: sensitivity, PPV: Positive Predictive Value; CI95%: Confidence Interval assuming a type I error of 5%

### Validity of HDAD to detect incident cases of prostate cancer

The most sensitive strategy (algorithm 2) was that which used diagnostic codes from position 1 to 5; whereas the most predictive (algorithm 4) (i.e. the highest PPV) was that which considered concurrent diagnoses and procedures from positions 1 to 5 (Table 2).

However, estimates for both Sensitivity and PPV were moderate to low. With regard to sensitivity, as expected, strategies based on diagnoses performed a little better than strategies based on concurrent diagnoses and procedures. However, except in ZD, first and second algorithm estimates scored under 25%. Although PPV performed better than sensitivity in BD and ZD (62% [CI95%: 55.6 to 68.0] in the former and 74.3% [CI95%: 67.0 to 80.6] in the latter), GD and MD included the 50% value (table 2).

### False negative and false positive underlying causes

#### With regard to the most sensitive algorithm

(Table 3), it should be noted that all datasets shared an important source of false negative cases, that is, those prostate cancer patients without admission (i.e. cases that were only treated as outpatients). In fact data ranged from 79% of cases in GD and MD to 39.3% in BD, with a 72.7% in ZD.

**Table 3:** Causes underlying positive and negative false cases in prostate cancer (with regard to the highest sensitive algorithm)

	Granada Dataset n (%)	Basque Country Dataset n (%)	Murcia Dataset n (%)	Zaragoza Dataset n (%)
**False negative cases**				

• Cases from private hospital in 2000	27 (14.5)	372 (42.7)	1 (0.3)	25 (9.3)
• Cases admitted in 2000 but discharged in 2001	3 (1.6)	98 (11.3)	41 (12.9)	43 (16.0)
• Cases coded in dx6 position or more	0 (0)	7 (0.8)	3 (0.9)	0 (0)
• False negative diagnoses	7 (3.8)	46 (5.3)	3 (0.9)	2 (0.7)
• Ambulatory care (instead of in-hospital care)	147 (79.0)	343 (39.3)	252 (79.7)	195(72.7)
• Loss using the algorithm	0 (0)	0 (0)	3(0.9)	0 (0)
• Other causes	2 (1.1)	4 (0.5)	13 (4.1)	3 (1.1)
	**186**(100)	**871**(100)	**316**(100)	**268**(100)

**False positive cases**				

• Cases registered in 2001	0 (0)	8 (2.3)	1 (0.8)	0 (0)
• Prevalent cases	56 (82.3)	280 (79.5)	74 (61.1)	122 (80.3)
• False positive diagnoses	7 (10.3)	15 (4.3)	29 (23.9)	25 (16.4)
• Missing in the registry	0 (0)	23 (6.5)	0 (0)	3 (1.9)
• Other residence than that covered by the registry	3 (4.4)	16 (4.5)	3 (2.5)	2 (1.3)
• Other causes	2 (2.9	10 (2.8)	14 (11.6)	0 (0)
	**68**(100)	**352**(100)	**121**(100)	**152**(100)

However, the main source of false negative cases in BD was private hospitalization (i.e. the lack of information from a set of hospitals without HDAD) during the period of study. This cause was responsible of 42.7% of BD false negatives. On the other hand, cases admitted in 2000 but discharged in 2001 were a relevant source of false negative cases in BD (11.3%), MD (12.9%) and ZD (16%).

Considering the false positive causes related to the most sensitive strategy, all datasets shared a very common cause; cases considered "incident cases" by the algorithm that actually were "prevalent cases" (data ranged form 61.1% in MD to 82.3% in GD). It should be noted that the following source of false positive cases was a coding error or clinical diagnosis misclassification in discharge records; this represented 4.3% in BD, 10.3% in GD, 16.4% in ZD and 23.9% in MD.

#### With regard to the most predictive algorithm

(Table 4), besides the already referred causes, an additional source of false negative cases was found: cases lost due to the restrictions of the algorithm itself. Data ranged from 11.8% of false cases in GD to 26.9% in ZD, suggesting coding differences among local HDADs.

**Table 4:** Causes underlying negative and positive false cases in prostate cancer (with regard to the highest predictive algorithm)

	Granada Dataset n (%)	Basque Country Dataset n (%)	Murcia Dataset n (%)	Zaragoza Dataset n (%)
**False negative cases**				

• Cases from private hospital in 2000	27 (12.8)	372 (37.4)	0 (0)	25 (6.8)
• Cases admitted in 2000 but discharged in 2001	3 (1.4)	98 (9.8)	42 (11.1)	43 (11.7)
• Cases coded in dx6 position or more	-	7 (0.7)	3 (0.8)	-
• False negative diagnoses	7 (3.3)	46 (4.6)	3 (0.8)	2 (0.5)
• Ambulatory care (instead of in-hospital care)	147 (69.7)	343 (34.4)	262(69.3)	195(53.1)
• Loss using the algorithm	25 (11.8)	125 (12.6)	52 (13.7)	99(26.9)
• Other causes	2 (0.9)	4 (0.4)	16 (4.2)	3 (0.8)
	**211 **(100)	**995**(100)	**378**(100)	**367**(100)

**False positive cases**				

• Cases registered in 2001	-	2 (2.1)	1 (3.8)	-
• Prevalent cases	9 (69.2)	78 (82.1)	14 (66.7)	36 (81.8)
• False positive diagnoses	4 (30.7)	-	4 (15.4)	6 (13.6)
• Missing in the registry	-	4 (4.2)	0 (0)	-
• Other residence than that covered by the registry	-	7 (7.4)	1 (3.8)	2 (4.5)
• Other causes	-	4 (4.2)	6 (23.1)	-
	**13**(100)	**95**(100)	**26**(100)	**44**(100)

On the other hand, incident cases which were actually prevalent were also the main cause of false positive cases in the most predictive algorithm (data ranged from 66.7% of false cases in MD to 82.1% in BD).

## Discussion

A linking-records-based validation study was carried out to determine whether the HDAD might be a useful tool to identify incident cases of prostate cancer. 2286 incident prostate cancer cases, recorded in four Spanish population-based cancer registries, were used to estimate its validity. Four algorithms were developed to detect incident cases. Those using only diagnostic codes were the most sensitive strategies although sensitivity scored around 25%; those using concurrent information about procedures were the most predictive, although positive predictive value scored close to 50%.

There is no recent literature available for comparison. Some international literature showed higher values for sensitivity [13] in prostate cancer, although the context was extremely different (cases attended 20 years ago, a completely different prostate cancer management, different use of administrative databases and a completely different healthcare system) to allow a reasonable discussion about the consistency of our results. Unfortunately, in our context, the only published study evaluated colorectal cancer [16].

Nevertheless, the analysis of false positive and false negative underlying causes has provided very useful insights. Three possible sources of failure to detect incident cases could be described in our study: the HDAD itself, the algorithms to detect cancer, and the features of this specific cancer site.

### Shortcomings for incident case detection using the HDAD

Firstly, although HDAD has been a compulsory information system for public institutions since the mid 90s in Spain, it was not mandatory for private hospitals. Thus, it must be expected that in those places where population is served by hospitals not required to provide hospital information, the capacity of HDAD to find cases when they exist in the registry will be limited.

In fact, this is the reason for the high number of false negative cases in the Basque Country, region in which the private-public mix is more influential in prostate cancer treatment (32% of prostate cancers registered the year under study were considered false negatives for this cause). In the remaining regions where this effect is expected to be less important, the percentage of false negative cases was lower: only 27 cases in Granada (12% of registered cancer), 25 cases in Zaragoza (5%) and 1 case in Murcia (0.3%) where information about private hospitals was also included.

Secondly, the population covered by a registry and the population attended by a hospital (from which the cases were retrieved) are different. Procedures like radical prostatectomy are usually delivered in high-volume hospitals, in which populations from different healthcare areas or regions are regularly referred to. These cases were identified as false positive cases because these patients belonged to a different area than that covered by the registry.

Although this was not an important source of false positive cases, in the most sensitive algorithm, figures ranged from 1.3% in ZD to 4.5% in BD.

Thirdly, coding processes seem to act as a potential source of variation within and among regional settings in different ways. In fact, empirical data show figures that deserve some comment. False positive diagnoses (i.e. HDAD registered prostate cancer when other type of cancer or even a benign tumour was registered in the registry) varied among regions. Taking into account the most sensitive definition, over all prostate cancer cases identified by HDAD, false positive diagnoses ranged from 4.3% in BD to 23.9% in MD (previous research found 7% of inappropriate use of codes [11]).

Another element that potentially could affect the number of false negative cases is the number of ICD codes registered for each patient, in each hospital, both in diagnoses and procedures. Slight differences have been found among registries: HDAD in Granada registered a mean of 4 diagnoses and 2 procedures; Basque Country recorded a mean of 3 diagnoses and 1 procedure, Murcia registered a mean of 5 diagnoses and 2.4 procedures and Zaragoza registered a mean of 4.2 diagnoses and 2.1 procedures. However, although a potential cause, the number of false negative cases for this reason was negligible.

### Shortcomings related with the algorithms

As expected [11,13] algorithms including concurrent procedures were more "restrictive", and their positive predictive values were higher than those without surgical codes. As a consequence of being so "specific" there was a significant loss of prostate cancer cases (i.e. false negative cases): 25 in GD, 125 in BD, 52 in MD and 99 in ZD. These numbers represented 11.8% of cases detected in GD, 12.6% in BD, 13.7% in MD and 26.9% in ZD and probably refers to those cases admitted for diagnostic tests that did not require surgical treatment.

On the other hand, a noticeable number of false negative cases were due to the fact that there were patients diagnosed in 2000 (i.e. admission to the hospital was in 2000) but they were discharged in 2001. This ranged from 1.4% of cases in GD to 11.7% of cases in ZD. However, flagging properly theses cases, for example by opening the timeframe two months forward, we would barely increase sensitivity in a 10%, remaining figures under 50%.

An additional source of false negative cases could be found in the exclusion of metastatic disease in the definition of incident case; it would the case for patients that initially present with metastases and the original prostatic tumour is only found once they are discharged. These patients would be flagged as false negatives if they are discharged without definitive diagnoses. None out of the 58 cases which could potentially be affected in our sample was actually misclassified.

Finally, although several strategies were used to reduce the number of prevalent cases (patients with an episode were followed backwards two years and specific exclusion criteria were used) a remarkable number of prevalent cases were retrieved by the algorithm. As expected the most specific strategy (algorithm 4) identified less prevalent cases than the most sensitive one (algorithm 2); however, 9(69.2%) prevalent cases were found in GD, 78 (82.1%) in BD, 14(66.7%) in MD and 36(81.8%) in ZD. When we reviewed cases registered in 1997 and earlier, we found a 48% of prevalent cases. This finding suggests that considering a timeframe backward of 5 years would be better in terms of gaining higher positive predictive values.

### Shortcomings related with the cancer site

Long survival cancers, like prostate cancer, are supposed to have more hospital readmissions than aggressive short survival cancers. Probably, the unexpected number of prevalent cases is related with us having decided to use too short a timeframe. In fact, in a ten-year period study Brackley *et al*. [22] found a rate of false positive as small as 1%, although authors recognised a significant overlapping between HDAD and registries.

But the most important element to be considered is the different manner in which healthcare services manage prostate cancer. In fact, 947 cases (41% of the total sample of prostate cancer registered by the 4 registries in 2000) were treated in ambulatory settings, being considered false negative cases by our methodology [147 cases in GD, 343 in BD, 262 in MD and 195 in ZD].

Thus, the more hospital-based the care provided the more probability of registering positive true cases by using the HDAD. This hypothesis was tested for breast cancer in a study that compared the Medicare and SEER registries agreement: concordance was considerably higher if surgery was performed (85% vs 50% when only biopsy was provided) [23].

Preliminary data from colorectal cancer and female breast cancer in Spain, two sites in which surgery is needed, show a better performance of positive predictive value: our context is consistent with this hypothesis: colorectal cancer and breast cancer showed a higher positive predictive value (up to 89% and up to 86%, respectively) [24-26].

Looking at these findings, we would be able to hypothesize that a good performance of our algorithms is expected in those cancers in which surgical treatment is needed and variations in surgical population rates are small (e.g. larynx, endometrial, colorectal or breast cancer). Conversely, those cancers with high uncertainty about the effectiveness of surgical intervention (e.g. prostate, oesophagus, pancreas, lung cancer) will perform poorly.

## Conclusions

Following the findings and discussion, in cancers like prostate cancer, we might distinguish some implications for cancer registries:

a) For automating the retrieval of incident cases, using algorithms combining diagnoses and procedures is a better option; however, opening the timeframe, being careful with the way cancer is registered, and properly recording the post-code where the patients live are important issues to be addressed.

b) On the other hand, some improvements should be implemented in the algorithms for cancer surveillance purposes. In this case, to widen the timeframe forward and to get cases from private institutions are important issues; however, the critical point is the need to combine inpatient and outpatient information in those cancers like prostate cancer, in which care is not only hospital-based.

HDAD seems to be a useful instrument to help cancer registries to reach their goals. Nevertheless, differences in the way cancers are managed across the healthcare system and the variability within and between registry procedures, suggest the need to be careful when HDAD is used.

## Competing interests

The authors declare that they have no competing interests.

## Authors' contributions

EBD, CM, MDC, CN, II, MJS & CM have been involved in the design, the analysis, the interpretation of results, and in the drafting process. NM & MM have programmed the linking record process and the STATA algorithms for detecting incident cases. CM, LH, JP, NL, OM, MCT, JB, MR, EP, YLC fixed databases for matching and checked every single case once the automatic linking process was finished. All authors read and approved the final manuscript.

## Pre-publication history

The pre-publication history for this paper can be accessed here:

http://www.biomedcentral.com/1472-6963/10/9/prepub

## References

[B1] Parkin D The evolution of the population-based cancer registry Nature Reviews Cancer 2006 6 603 612 10.1038/nrc1948 16862191

[B2] Penberthy L McClish D Pugh A Smith W Manning C Retchin S Using hospital discharge files to enhance cancer surveillance Am J Epidemiol 2003 158 1 27 34 10.1093/aje/kwg108 12835284

[B3] Black RJ Simonato L Storm HH Demaret E Automated data collection in cancer registration IARC Technical reports 1998

[B4] Tagliablue G Maghini A Fabiano S Tittarelli A Frassoldi E Costa E Nobile S Codazzi T Crosignani P Tessandori R Contiero P Consistency and accuracy of diagnostic cancer codes generated by automated registration: comparison with manual registration Population Health Metrics 2006 4 10 10.1186/1478-7954-4-10 17007640 PMC1592124

[B5] Brooks JM Chrischilles E Scott S Ritho J Chen-Hardee S Information gained from linking SEER Cancer Registry Data to state-level hospital discharge abstracts: Surveillance, Epidemiology, and End Results Med Care 2000 38 11 1131 40 10.1097/00005650-200011000-00007 11078053

[B6] Freeman JL Zhang D Freeman DH Goodwin JS An approach to identifying incident breast cancer cases using Medicare Claims data J Clin Epidemiol 2000 53 605 14 10.1016/S0895-4356(99)00173-0 10880779

[B7] Du X Freeman JL Warren JL Nattinger AB Zhang D Goodwin JS Accuracy and completeness of Medicare claims data for surgical treatment of breast cancer Med Care 2000 38 7 719 27 10.1097/00005650-200007000-00004 10901355

[B8] Cooper GS Yuan Z Jethva RN Rimm AA Use of Medicare claims data to measure county-level variation in breast carcinoma incidence and mammography rates Cancer Detect Prev 2002 26 3 197 202 10.1016/S0361-090X(02)00056-9 12269766

[B9] Rolnick SJ Hart G Barton MB Herrinton L Flores SK Paulsen KJ Husson G Harris EL Geiger AM Elmore JG Fletcher SW Comparing breast cancer case identification using HMO computerized diagnostic data and SEER data Am J Manag Care 2004 10 4 257 62 15124502

[B10] Koroukian SM Cooper GS Rimm AA Ability of Medicaid Claims Data to identify incident cases of breast cancer in the Ohio Medical Population Health Ser Res 2003 38 947 60 10.1111/1475-6773.00155 PMC1360924 12822920

[B11] Leung KM Hasan AG Rees KS Parker RG Legorreta AP Patients with newly diagnosed carcinoma of the breast: validation of a claim-based identification algorithm J Clin Epidemiol 1999 52 57 64 10.1016/S0895-4356(98)00143-7 9973074

[B12] Warren JL Feuer E Potosky AL Riley GF Lynch CF Use of Medicare Hospital and Physician Data to Assess breast Cancer Incidence Med Care 1999 37 445 56 10.1097/00005650-199905000-00004 10335747

[B13] Cooper GC Yuan Z Stange KC Dennos LK Amino SB Rimm AA The sensitivity of Medicare Claims Data for case ascertainment of six common cancers Med Care 1999 37 436 44 10.1097/00005650-199905000-00003 10335746

[B14] Couris CM Schott AM Ecochard R Morgon E Colin C A literature review to assess the use of claims databases in identifying incident cancer cases Health Services & Outcomes Research Methodology 2003 4 49 63 10.1023/A:1025828911298

[B15] Baldi I Vicari P Di Cuonzo D Zanetti R Pagano E Rosato R Sacerdote C Segnan N Merletti F Ciccone G A high positive predictive value algorithm using hospital administrative data identified incident cancer cases J Clin Epidemiol 2008 61 4 373 9 10.1016/j.jclinepi.2007.05.017 18313562

[B16] Márquez M Valera I Chirlaque MD Tortosa J Párraga E Navarro C Validación de los códigos diagnósticos de cáncer de colon y recto del Conjunto Mínimo Básico de Datos Gac Sanit 2006 20 4 266 72 10.1157/13091140 16942712

[B17] National Institute for Health and Clinical Excellence Prostate cancer: diagnosis and treatment 2008 London: National Institute for Health and Clinical Excellence

[B18] Oliva G Allepuz A Kotzeva A Tebé C Bernal-Delgado E Peiró S on behalf of the Atlas VPM Group Variaciones en hospitalizaciones por cirugía oncológica en el Sistema Nacional de Salud Atlas Var Pract Med Sistema Nacional de Salud 2009 3 2 241 72

[B19] Curado MP Edwards B Shin HR Storm H Ferlay J Heanue M Boyle P eds Cancer Incidence in Five Continents,160 Lyon, IARC 2007 IX IARC Scientific Publications No. 160, Lyon IARC

[B20] Márquez M Chirlaque MD Navarro C The DataLink Record Linkage Software Applied to the Cancer Registry of Murcia, Spain Meth Inform Med 2008 47 5 448 53 18852919 10.3414/me0529

[B21] Binomial exact confidence intervals ©Copyright 1996-2007 StataCorp LP. Accessed on 2008, March 18th http://www.stata.com/help.cgi?contents

[B22] Brackley ME Penning MJ Lesperance ML In the absence of cancer registry data, is it sensible to assess incidence using hospital separation records? International Journal for Equity in Health 2006 5 12 10.1186/1475-9276-5-12 17026764 PMC1613240

[B23] Cooper G Yuang Z Stange KC Denise LK Amini SB Rimm AA Agreement of Medicare Claims and Cancer Registry Data from Assessment of Cancer-related treatment Med Care 2000 38 411 21 10.1097/00005650-200004000-00008 10752973

[B24] Martos MC Chirlaque MD Bernal E Martínez C Izarzugaza I Martínez N Márquez M Larrañaga N Sánchez MJ Identifying incident cases of colorectal cancer from hospital discharge register in Spain XXXII Réunion des Registres des pays de Langue Latine Montreal 16-18 mai 2007 http://www.grellnet.org/2007/programme_montreal_v4_acces.pdf Accesed on 2008, March 18^th^

[B25] Chirlaque MD Martos M Izarzugaza I Martínez C Bernal E Martínez N Márquez M Larrañaga N Navarro C Validity of hospital discharge with diagnostic and procedures codes related to female breast cancer in Spain XXXII Réunion des Registres des pays de Langue Latine Montreal 16-18 mai 2007 http://www.grellnet.org/2007/programme_montreal_v4_acces.pdf Accesed on 2008, March 18^th ^

[B26] Izarzugaza MI Mokoroa O Larrañaga N Bidaurrazaga J Tobalina MC Bernal E Hospital discharge records registry as a tool to identify cancer incident cases in the Basque Country Programme and Book of Abstracts. 29th Annual Meeting of the International Association of Cancer Registries. At the crossroad of tradition and new technologies in cancer registration. Ljubljana, Slovenia 2007 46

